# Coupling epidemiological and tree growth models to control fungal diseases spread in fruit orchards

**DOI:** 10.1038/s41598-019-44898-6

**Published:** 2019-06-11

**Authors:** Daniele Bevacqua, Michel Génard, Françoise Lescourret, Davide Martinetti, Gilles Vercambre, Pierre Valsesia, Josè Manuel Mirás-Avalos

**Affiliations:** 1 UR 1115, Plantes et Systèmes de Culture Horticoles, Institut National de la Recherche Agronomique, Avignon, France; 2 UR 546, Bistatistique et Processus Spatiaux, Institut National de la Recherche Agronomique, Avignon, France

**Keywords:** Agroecology, Drought

## Abstract

Agronomic practices can alter plant susceptibility to diseases and represent a promising alternative to the use of pesticides. Yet, they also alter crop quality and quantity so that the evaluation of their efficacy is not straightforward. Here we couple a compartmental epidemiological model for brown rot diffusion in fruit orchards with a fruit-tree growth model explicitly considering the role of agronomic practices over fruit quality. The new modelling framework permits us to evaluate, in terms of quantity and quality of the fruit production, management scenarios characterized by different levels of regulated deficit irrigation and crop load. Our results suggest that a moderate water stress in the final weeks of fruit development and a moderate fruit load provide effective control on the brown rot spreading, and eventually guarantee monetary returns similar to those that would be obtained in the absence of the disease.

## Introduction

Plant diseases are responsible for important crop losses worldwide and represent a threat to global food security^1^. Disease control mostly relies on chemicals, with inherent environmental costs, and the interest in finding alternative ways for crop protection is increasing. Advances in epidemiology of crop disease shows that some agricultural practices, which do not involve the use of chemicals (e.g. irrigation, shoot pruning, fruit thinning), can alter the vulnerability of the host and the environment in which diseases develop, and eventually control the epidemic^2^. However, agricultural practices also alter the quality and quantity of the final yield and the evaluation of their efficacy should therefore consider both their consequences on the disease outbreaks and on the yield quantity and quality. Although the main concern with crop diseases is the yield loss, this component is often neglected in epidemiological models while those crop models that focus on yield estimate, usually disregard epidemic dynamics^3,4^.

Brown rot caused by *Monilinia* spp. is one of the most serious diseases of stone fruit. It affects species with important economic value such as peach, plum, apricot, cherry, and almond and it is present in all temperate regions^5^. It is commonly controlled using fungicides whose efficacy decays in time, due to the emergence of fungicide resistant strains^6,7^. Peach (*Prunus persica*) is a stone fruit plant with important economic value, cultivated in continental and temperate climates worldwide, and whose physiology was intensively studied by means of crop and fruit physiology models in the last decades^8–12^. Regulated Deficit Irrigation (RDI) is an agronomic practice intended to reduce water use without compromising the yield by reducing irrigation during those stages of fruit development that are less sensitive to water stress. Such a practice is widely used in those countries where water shortage is becoming an issue^13–16^ and recent studies investigated its consequences on yield quantity and quality, and on pest and disease control^17–21^. Fruit thinning is an effective and common agronomic practice to reduce the crop load (CL) in fruit trees^22^. Crop load affects the relationship between carbon “sink” and “source” plant organs and its reduction enhances the average fruit mass, overcomes alternate bearing, increases nutrient accumulation and prevents premature tree aging^23^. Moreover, some works investigated the possible role of fruit thinning in controlling fungal disease by altering the host (fruit) susceptibility and density^24–26^.

In a recent work, Bevacqua *et al*.^27^ proposed a Susceptible Exposed Infectious (SEI) model to describe the temporal dynamics of brown rot spreading in fruit orchards and evaluate the consequent marketable yield. They found that CL has important consequences on the progression of brown rot epidemics. This model allowed simulating the response of the marketable yield to different CL levels. However, fruit quality, which includes both commercial size norms (e.g. minimum fruit size) and organoleptic criteria (e.g. color, firmness, sugar concentrations), is also an important aspect of fruit production^28^ and varies with CL^29^. The fruit-tree growth model QualiTree^30^ permits to simulate the effect of various agricultural practices on the average fruit size and quality. QualiTree was successfully used to describe the consequences of CL and RDI on different peach cultivars^17,31^. It reliably simulated that low CLs decrease total yield but increase average fruit size at harvest^17^ and that RDI decreased average fruit size while increasing its sugar content. Also, QualiTree showed that the effect of water stress on the yield depends on the period of fruit growth at which it occurs^31^. In the present work, we couple the SEI model, mentioned above, with QualiTree to evaluate the consequences of different combinations of CL and RDI over the quantity and quality of fruit yield in the prospect of a possible spreading disease. We use the brown rot – peach as model pathosystem. The resulting modeling framework permits to estimate the overall yield mass, which represents yield quantity, and the average fruit size and sweetness, which determine yield quality. Eventually, as yield quantity and quality differently responded to different management scenarios, in the presence or absence of the disease, we performed a multi-criteria analysis (MCA) by means of a Pareto analysis and a simple, yet based on widely accepted principles, monetary valuation to rank the different simulated scenarios and explore the consequences of different management scenarios on the estimated economic returns.

## Results

**Figure 1:**
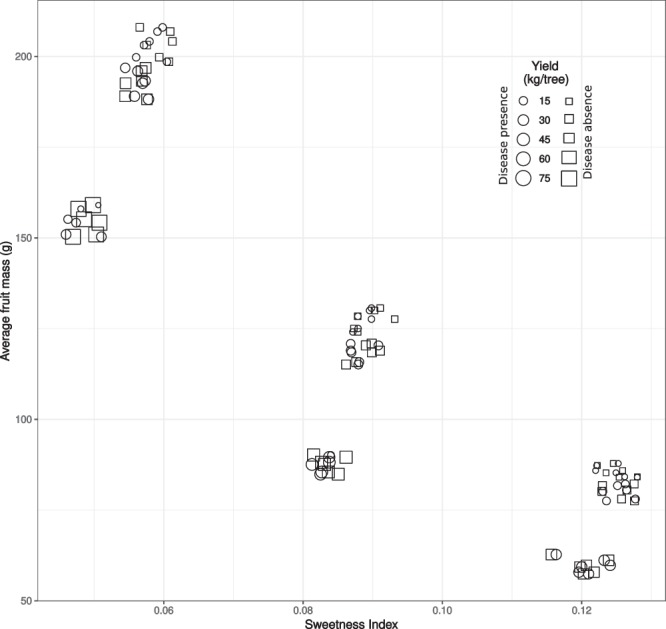
Expected performances of the 54 considered scenarios, in the presence and absence of the disease, with respect to three different management objectives: maximizing total yield mass, average fruit mass, fruit sweetness index. (“jitter” function was used to separate overlapping points and increase legibility).

**Figure 2:**
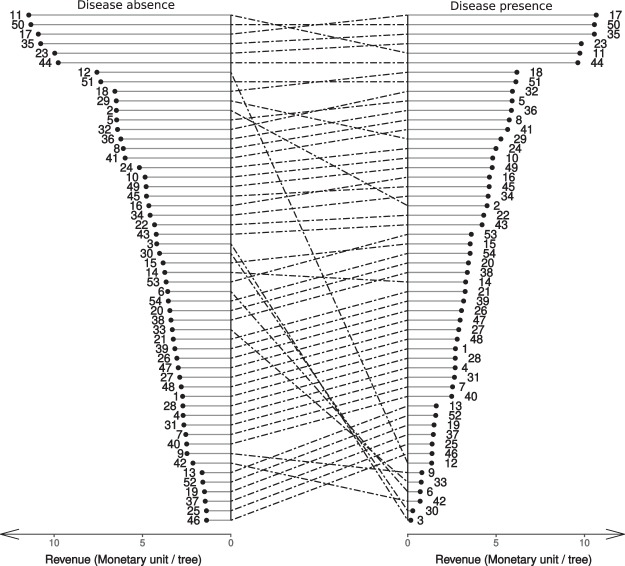
Expected performances of the 54 considered scenarios, in the presence and absence of the disease, with respect to monetary revenue.

The simulated yield in the different *CL* × *RDI* management scenarios, in absence and presence of brown rot disease, its composition into susceptible, exposed and infected fruit, the average fruit mass and sweetness index are entirely reported as supplementary information (Table 1) and summarized in Fig. 1. Depending on the management scenario, the yield varies between 4 (4)–58 (75) kg plant^−1^ in presence (absence) of the disease, the average fruit fresh mass between 57–208 g and the sweetness index between 5–13%. Note that fruit mass and sweetness determined by QualiTree, irrespective of the presence (absence) of the disease, are negatively correlated (correlation coefficient = −0.87). With the unique objective of maximizing the overall yield mass, the best management scenario, in absence of disease, would be high CL and low RDI in all the three considered periods of fruit development (MS = 3, in Table 1). This would imply a yield of 75 kg tree^−1^ composed by fruit with an average mass of 159 g and 5% of sweetness index. On the other hand, when the disease is present, the highest yield would be obtained with high CL, low and moderate RDI, respectively in the first and in the following two fruit growth periods (MS = 18, in Table 1). Such a management would result in a yield of 39 kg tree^−1^ with fruit of 88 g and a sugar index of ca. 8%. The maximum fruit mass (208 g) is obtained for low CL and low RDI in any fruit growth period (MS = 1, Table 1) while the maximum sweetness (ca. 13%) is obtained for low CL and high RDI in any fruit growth period (MS = 46, Table 1). Pareto front regarding the maximization of three concurrent objectives (i.e. maximization of overall yield, fruit size and fruit sweetness) is composed by a multitude of scenarios (MS = 1, 2, 3, 4, 5, 6, 10, 11, 12, 13, 14, 15, 21, 25, 28, 39, 46, 50, 51, 52, 53, 54 in absence of the disease and MS = 1, 2, 4, 5, 8, 10, 11, 13, 14, 15, 17, 18, 21, 24, 25, 32, 39, 46, 49, 50, 51, 52, 53, 54 in the presence of the disease) indicating that these are contrasting objectives. In other words, it is not possible to maximize one, without compromising the other two, being impossible to determine one best scenario. This issue is overtaken by means of Eqs 6–8 which provide a monetary value per unit mass of the yield as a function of the average fruit mass and sweetness, which, together with the estimate of yield quantity, allows to ascribe an expected revenue to any management scenario (Fig. 2) and thus to rank them accordingly.

The maximum expected revenue in absence of the disease (11.4 § plant^−1^, MS = 11, § denoting an arbitrary monetary unit) is obtained for moderate CL, low RDI in 1^st^ and 2^nd^ fruit growth periods and moderate RDI in the 3^rd^ one. Yet, such a management scenario would would be ranked as 5^th^ in presence of the disease as it would produce a revenue of 9.7 § plant^−1^. On the other hand, in presence of the disease one could obtain similar revenues (>10 § plant^−1^) in a number of other scenarios (Fig. 2; MSs = 17, 50, 35) characterized by moderate CL, different combinations of RDI in 1^st^ and 2^nd^ fruit growth periods and moderate RDI in the 3^rd^ one. Those scenarios (MSs = 3, 6, 12, 30, 33) whose rank dramatically drop (i.e. more than 10 positions lost) when passing from absence to presence of the disease are all characterized by high CL and low/moderate RDI in all the growth periods.

**Figure 3:**
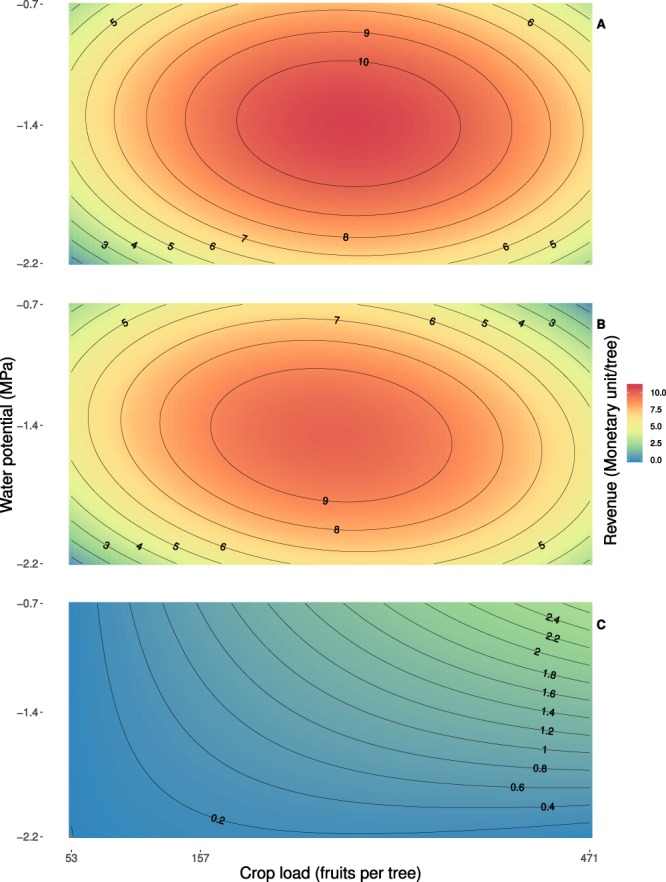
Estimated surfaces showing expected monetary revenue in the absence of the disease (**A**), in the presence of the disease (**B**), monetary losses due to the disease (**C**), as a function of initial crop load CL and water potential in the last five weeks of fruit growth.

Response Surface Methodology, RSM, indicate that the expected revenues, in both presence and absence of the disease, are mainly affected by CL and RDI in the 3^rd^ considered growth period and can be effectively fitted (*R*^2^ = 0.86 and 0.85, respectively in absence and presence of the disease) with the second-order equation:

1 $$\hat{R}=a+b\,CL+c\,RD{I}_{{\rm{3}}}+d\,CL\times RD{I}_{{\rm{3}}}+e\,C{L}^{{\rm{2}}}+f\,RD{I}_{{\rm{3}}}^{{\rm{2}}}$$

where *a* = 9.32 (8.83), *b* = 8.18 (6.85), *c* = 0.36 (−0.01), *d* = −0.71 (−1.76), *e* = −10.73 (−10.36), *f* = −4.27 (−4.27), in absence (presence) of the disease. Revenues response surfaces are reported in Fig. 3. They show that (*i*) revenues are maximized for CL between 250–300 fruit per tree and moderate RDI in the last five weeks of fruit growth; (*ii*) losses due to the presence of the disease can be limited through a sound combination of agronomic practices and are maximal at high CL and absence of water stress in the last five weeks of fruit growth (Fig. 3C).

## Discussion

To our knowledge, the present work represents the first attempt to couple an epidemiological model, considering basic principles for the transmission of infection between infected and susceptible individuals, with a crop model that estimates yield quantity and quality while explicitly accounting for agronomic practices. In literature, a robust theoretical framework for the analysis and control of plant disease epidemics is available^32^. Theoretical works usually deal with concepts of invasion and persistence of the pathogen in a host population^33–35^. They provide excellent insights on the system functioning by showing, for instance, for which combinations of epidemiological parameters (e.g. infectious period and transmission rate) the invasion is possible. Yet, the transfer of theoretical findings into agronomic practices is far from being straightforward since (*i*) the relationship between agronomic practices and model components (e.g. parameters, initial state variables) is often neglected; (*ii*) the timespan of a growing season is rarely suited to asymptotic analyses, which are generally used in theoretical studies; and (*iii*) the objective of the growers is to maximize the yield rather than to impair pathogen invasion *per se*. From an agronomic point of view, it makes no sense to reduce crop size below a critical value to impair pathogen invasion, if this impairs also the final yield. According to Cunniffe *et al*.^3^, “models should incorporate sufficient epidemiological realism in order to analyse and predict the effects of disease and host dynamics on yield”. We claim that sufficient realism regarding plant physiology should also be incorporated to predict the effect of disease on the yield, and we are convinced that the present work goes in this direction.

Our results indicate that agronomic practices based on regulated deficit irrigation and fruit thinning can provide effective control on the brown rot spreading, and eventually guarantee monetary returns similar to those that would be obtained in the absence of the disease, a situation that is actually achieved through the use of fungicides. Previous field observations indicated that agronomic practices play a role in the diffusion of brown rot: water deprivation and open vase training with centrifugal pruning were associated to lower brown rot incidence^25,36–38^. Our modelling approach can help to identify which epidemiological processes are responsible for the observed patterns, and optimize the deployment of control strategies. Our work indicates that the considered agronomic practices (fruit thinning and regulated deficit irrigation) affect brown rot diffusion as they affect fruit densities, which is proportional to the rate of exposure to the pathogen, and fruit growth, which determines fruit skin integrity and therefore fruit probability to get infected, once it is exposed to the pathogen. Pareto analysis clearly shows the trade-off between different objectives and let the decision maker to choose between a set of optimal (i.e. non-dominated) policies. On the other hand, the use of a monetary valuation allows deriving a formal ranking of the different scenarios. Our naive monetary valuation points out the fact that fruit quality is an important issue to be considered when evaluating agronomic strategies as it determines economic returns that eventually drive growers behavior. Our results indicate that high and moderate CLs are likely to maximize economic returns, respectively in absence and presence of the disease. If we considered a cost for fruit harvest, which increases with CL, and the fact that high CL in a given season might impair the yield of the following one (which is common in perennial fruit trees^23,39^), the choice of a moderate CL would turn out to be the best one, irrespective of the presence of the disease. One could argue that also fruit thinning has a cost, however it is much lower than that of fruit harvest which requires much more attention and consequent fruit storage. Thus, the actual habit of peach growers of applying a moderate crop load turns out to be an economically optimal choice. Our results indicate that RDI affects revenues when it is applied to the last five weeks of fruit growth. In fact, in this period, fruit significantly increases in mass and sugar contents^37,40^ and if on one hand, a minimal irrigation level is required to make fruit attaining a marketable size, on the other hand a minimal water stress is required to increase sugar concentrations. The fact that growers usually apply restriction irrigation in the previous pit hardening stage^14,37^ is because in that stage, the fruit growth is less sensitive to water restriction. However, our results suggest that it would be more effective to provide a moderate water stress in the last stage of fruit pulp growth rather than during pit hardening. In fact, our results suggest that a moderate water restriction should not be applied in those development stages in which its effect on the fruit would be irrelevant (with the sole aim of saving water) but in that period in which it effectively affects the fruit development to provide fruit that is less sensitive to brown rot infection and with higher quality. Our results are in agreement with field works showing that, with the aim of reducing brown rot incidence, water deprivation is more efficient when applied during the last weeks of pulp growth^36–38^. One of the key findings of the present work is that, when the scenarios are ranked according to a monetary valuation, the revenues are not dramatically impaired in the presence of the disease, if a sound management is adopted. A number of scenarios would allow for more than 10 § tree^−1^ that is not far from the maximum value of 12.9 § tree^−1^ obtainable in the absence of the disease. Moreover, considering that fungicide application and irrigation have a cost, and that the willingness to pay of the consumers might increase for fruit issued from sustainable agronomic practices that minimize the use of fungicides and water, we might have underestimated the revenues in the presence of the disease.

## Methods

### The SEI model

The SEI model presented in Bevacqua *et al*.^27^ describes the temporal dynamics of a population composed by host fruit in a given orchard, during the growth stage that starts at time *t*_0_, following fruit thinning, and ends at the harvest time, *t*_H_, in relation to brown rot infection. Growing fruit can be susceptible (*S*), exposed to the pathogen (*E*), or infected and infectious (*I*). The model assumes that (*i*) the probability per time unit that a susceptible fruit is exposed to the pathogen is proportional to the density of infectious fruit and that (*ii*) exposed fruit progresses to the infectious state at a rate that is proportional to the cuticle crack surface area^26^, which itself varies in time. Cuticle cracks start appearing when the fruit surpasses a critical size and, thereafter, the cuticle crack surface is proportional to the fruit mass^41^.

According to the model outline and assumptions, the epidemics, from *t*_0_ to *t*_H_, are described with the following system of ordinary differential equations.

2 $$\begin{array}{c}\frac{dS}{dt}=\eta \,E-\lambda \,I\cdot S\\ \frac{dE}{dt}=\lambda \,I\cdot S-\eta \,E-\sigma (t)\cdot E\\ \frac{dI}{dt}=\sigma (t)\cdot E-\rho I\end{array}$$

where *t* represents the time, *η* represents the rate at which the spores deposited on the fruit surface die and the fruit returns susceptible, *λ* represents the disease transmission rate per infectious unit, *σ*(t) represents the rate at which a fruit exposed to the pathogen transfers into the infectious compartment, and *ρ* represents the death rate of an infectious fruit. According to Gibert *et al*.^41^,

3 $$\sigma (t)=\begin{array}{cc}0 & {\rm{i}}{\rm{f}}\,m(t) < {m}_{C}\\ \gamma \cdot m(t) & {\rm{i}}{\rm{f}}\,m(t) > {m}_{C}\end{array}$$

where *γ* is an infection constant, *m*_c_ is the fruit mass threshold for cuticle cracking and *m*(t) describes the increase of the fruit fresh mass during the growing season. Details on the parameter values can be find in the original description of the model^27^. In the original version of the model, fruit mass varied in time according to a logistic equation. Yet, since agronomic practices have important consequences on the temporal variation of fruit mass, this can be more realistically simulated by a fruit-tree model explicitly accounting for agronomic practices.

### QualiTree

QualiTree^30^ is a generic fruit-tree model that considers the tree as a set of objects: some compartments viewed globally such as old wood (trunk and branches), water sprouts, and roots (coarse and fine), and some viewed in more detail and placed in the tree architecture, such as fruiting units (FUs). The FUs are composed of fruits, leafy shoots, and stem wood. The tree is virtually regarded as a collection of FUs connected to old wood within an explicit architecture (topology and geometry). A simple module for estimating canopy radiation interception over the growing season is used to simulate the photosynthesis and transpiration on each FU. In QualiTree (see Lescourret *et al*.^30^ for the core of the model, Mirás-Avalos *et al*.^42^ for the associated light interception model and Rahmati *et al*.^21^ for energy balance calculation and water transfer formalization within the plant), the carbon processes are assimilation, exchange within the tree, utilization for organ growth, mobilization and replenishment of reserves, and metabolic transformation of various sugars in the fruit. Water processes are transpiration, flow within the tree and fruit water inflows and outflows. Rather than adopting a single view, QualiTree uses several approaches for carbon partitioning within the tree. At the tree level, QualiTree uses a teleonomic approach by considering, based on the root-shoot functional balance principle, that the tree tends to restore the C balance between leafy shoots and fine roots. QualiTree also uses source-sink approaches to represent the effect of thinning that removes sinks (fruits) but also to represent carbon transport within the tree. After satisfaction of maintenance respiration requirements for every tree organ, flows are calculated between “source” (*i*) and “sink” (*j*) tree entities based on the available carbon in *i*, the carbon demand in* j* and the *i-j* distance. Water flow within plant three-dimensional architecture is described by laws that connect the different axes through their hydraulic conductivity and by their water potential. Leaf, stem and fruit water potential are outputs of the energy balance model linked to the hydraulic transfer model. Finally, the accumulation of water in the fruit is simulated as a consequence of turgor pressure effect on cell expansion. QualiTree addresses a broad range of fruit quality traits: fruit size, proportion of flesh in the total fruit mass, dry matter content of the flesh, concentrations of various sugars in the flesh, and sweetness. Fruit sugar content is described by a set of differential equations that accounts for the three processes involved in the development of soluble sugars within the fruit: sugar importation, metabolism and water dilution. The key interest of coupling Qualitree and the SEI epidemiological model is that QualiTree permits to simulate, for any agronomic scenario, the temporal trajectories of the single fruit mass, *m*(*t*), and a broad range of quality traits including a sweetness index, *s*(*t*); this latests obtained from a linear and weighted combination of the different sugar concentrations^30^.

### Model coupling and agronomic scenarios

The coupling of the two models consists in running the SEI model by using the temporal fruit mass trajectories, *m*(t), originated by QualiTree for a given management scenario (MS). We defined an agronomic scenario by a level for any of the four considered agronomic factors: crop load (CL) following fruit thinning, regulated deficit irrigation (RDI) in a first (i.e. 75–91 days after bloom DAB), a second (i.e. 92–105 DAB) and a third (106–140 DAB) fruit growth period. Time and duration of different fruit development stages depends on the cultivar variety. We used a mid-late maturing variety (i.e. ‘Suncrest’)^42^ and the three considered periods respectively represent the pit hardening stage, the initial and final phase of the pulp extension stage. For crop load, we considered three possible levels: low (53 fruits tree^−1^), moderate (157 fruits tree^−1^) and high (471 fruits tree^−1^). The crop load determined by fruit thinning determines the fruit abundance of the SEI model (i.e. S + E + I) at initial time *t*_0_ and affects the fruit mass curves *m*(*t*) that are produced by QualiTree. Accordingly to previous works^27,43^, we set *S*(*t*_0_) and *E*(*t*_0_) respectively equal 0.3% and 1.8% of the entire fruit population. For the RDI, we considered different levels of water stress during each considered fruit growth period. We express the level of RDI as the average minimum daily root collar potential, Ψ_r_. We used reference values of minimum Ψ_r_ estimated in previous works on a mid-late maturing peach cultivar ‘Catherine’^44,45^. We derived hourly values of water potential assuming a sinusoidal variation, during the day, with an amplitude determined by a unique maximum water potential (Ψ_r_ = −0.3), measured before dawn, and a minimum, measured at midday, water potential with three different levels: −0.7, −1.4 and −2.2 Mpa respectively for low (absent), moderate and high water stress.

The combinations of the different levels of crop load and water restrictions determined 54 possible management scenarios (Supplementary Information Table 1). For each scenario we derived the initial abundance of fruit in the SEI model and a trajectory of *m*(*t*) and *s*(*t*). We ran the SEI model to obtain the density of fruit in the *S* and *E* classes, which eventually, at harvest time *t*_h_, determine the marketable yield. Growers usually harvest peach fruit when it attends a convenient marketable size (e.g. 80 g) and it stops growing (i.e. its relative growth rate RGR < 0.008 d^−1^). Yet, if these conditions are not met, they harvest the fruit at a limit date *t*_MAX._ = 6^th^ August. Eventually, for any agronomic scenario we estimate the yield quantity (Y) as:

4 $$Y=(S({t}_{H})+E({t}_{H}))\cdot m({t}_{H})$$

while its quality is related to average fruit mass *m*(*t*_H_) and sweetness *s*(*t*_H_).

### Multi-criteria analysis and monetary evaluation

The objective of growers is to maximize the yield quantity and quality. To highlight possible trade-offs between these objectives and to determine the management policies resulting in the best compromise between them, we performed a multi-criteria analysis (MCA), which provides a useful framework to develop realistic management policies when conflicting objectives are considered^46^ and it has been used to rationalize the management of a number of crops^47,48^. Following the classical theory for MCA, we identified the Pareto-efficient alternatives, i.e. the management policies for which it is not possible to modify decision variables to improve one performance indicator (for instance, the yield quantity) without worsening at the same time the other performance indicator (i.e. single fruit size). The non-dominated policies identify the so-called Pareto front. Although such a front and associated trade-offs supply a useful reference and important information to decision makers, they do not point out a best scenario as result of a formal maximization problem. On the other hand, a monetary evaluation, which consists in reducing the problem to a single objective by giving a monetary value to the different objectives, permits to obtain a formal solution of the optimization problem. In our particular case, this analysis is suited since the producers are not interested in the fruit quality *per se* but because it is related to the fruit monetary value. In fact, the fruit value is negligible for fruit below a minimum size, *m*_MIN_, or sugar content, *s*_MIN_; it increases with both *m* and *s* and saturates at a maximum monetary value^49,50^. We then expressed the dependence the fruit value to *s* and *m* through a logistic function with values between 0 and 1:

5 $$f(x)={({\rm{1}}+{e}^{{\eta }_{x}({\lambda }_{x}-x)})}^{-{\rm{1}}}$$

where *x* can be fruit size (x = *m)* or sweetness index (x = *s)*, *η*_x_ is the consumer sensitivity to the variable *x*, and *λ*_x_ is a semi-saturation value for the variable *x*. According to arbitrary, yet realistic, consumer preferences we set *λ*_m_ = 100 g, *η*_m_ = 0.05, *λ*_s_ = 7%, *η*_s_ = 98.1. This is equivalent to say that the monetary value of a fruit unit equals 50% of its potential for *m* = 100 g or *s* = 7%, and it attains 95% of its potential for *m* = 160 g or *s* = 10%. We then used the counter-monotonic copula corresponding to the lower-bound of the Fréchet-Hoeffding copula theorem^51^ to compute the monetary value of a unit of fruit as a function of both its mass and sweetness:

6 $$MV=max({\rm{0}},f(m)+f(s)-{\rm{1}})$$

Such a copula is particularly suited to compute the bivariate probability distribution of two variables that show a negative correlation, as it is the case for fruit sweetness and mass (the bigger the fruit, the lower the sweetness index, correlation = −0.87). Eventually, the expected monetary revenue per tree *R*, under any agronomic scenario, can be easily computed as

7 $$R=Y\cdot MV$$

In order to explore the relationships between the considered agronomic practices and and the expected revenue, *R*, we used the Response Surface Methodology, RSM^52^ with a second degree polynomial model.

## Supplementary information

Results-Table

## References

[1] Strange RN, Scott PR (2005). Plant Disease: A Threat to Global Food Security. Annu. Rev. Phytopathol..

[2] van Bruggen AHC, Finckh MR (2016). Plant Diseases and Management Approaches in Organic Farming Systems. Annu. Rev. Phytopathol..

[3] Cunniffe N (2015). Thirteen challenges in modelling plant diseases. Epidemics.

[4] Savary S, Teng PS, Willocquet L, Nutter FW (2006). Quantification and Modeling of Crop Losses: A Review of Purposes. Annu. Rev. Phytopathol..

[5] Oliveira Lino L (2016). Brown Rot Strikes Prunus Fruit: An Ancient Fight Almost Always Lost. J. Agric. Food Chem..

[6] Thomidis T, Michailides T, Exadaktylou E (2009). Contribution of pathogens to peach fruit rot in northern Greece and their sensitivity to iprodione, carbendazim, thiophanate-methyl and tebuconazole fungicides. J. Phytopathol..

[7] Zhu X, Chen X, Guo L (2011). Population Structure of Brown Rot Fungi on Stone Fruits in China. Plant Dis..

[8] Grossman YL, DeJong TM (1994). PEACH: A simulation model of reproductive and vegetative growth in peach trees. Tree Physiol..

[9] De Jong TM, Goudriaan J (1989). Modelling peach fruit growth and carbohydrate requirements:reevaluation of the double-sigmoid growth pattern. J. Amer.Soc.Hort.Sci.

[10] Lescourret F, Génard M (2005). A virtual peach fruit model simulating changes in fruit quality during the final stage of fruit growth. Tree Physiol..

[11] Lescourret F, Ben Mimoun M, Génard M (1998). A simulation model of growth at the shoot-bearing fruit level I. Description and parameterization for peach. Eur. J. Agron..

[12] Génard M, Pagès L, Kervella J (1998). A carbon balance model of peach tree growth and development for studying the pruning response. Tree Physiol..

[13] Naor A (2001). The Response of Nectarine Fruit Size and Midday Stem Water Potential to Irrigation Level in Stage III and Crop Load. J. Am. Soc. Hortic. Sci..

[14] Girona J (2003). Peach Tree Response to Single and Combined Regulated Deficit Irrigation Regimes under Shallow Soils. J. Am. Soc. Hortic. Sci..

[15] Bryla DR (2005). Influence of irrigation method and scheduling on patterns of soil and tree water status and its relation to yield and fruit quality in peach. HortScience.

[16] Ruiz-Sanchez MC, Domingo R, Castel JR (2010). Review. Deficit irrigation in fruit trees and vines in Spain. Spanish J. Agric. Res..

[17] Mirás-Avalos JM (2013). Combined effects of water stress and fruit thinning on fruit and vegetative growth of a very early-maturing peach cultivar: Assessment by means of a fruit tree model, QualiTree. Irrig. Sci..

[18] Koricheva J, Larsson S, Haukioja E (1998). Insect Performance on Experimentally Stressed Woody Plants: A Meta-Analysis. Annu. Rev. Entomol..

[19] Rousselin A (2018). Rosy apple aphid abundance on apple is shaped by vegetative growth and water status. Crop Prot..

[20] Rousselin A (2016). Nitrogen and water supplies affect peach tree-green peach aphid interactions: the key role played by vegetative growth. Agric. For. Entomol..

[21] Rahmati, M. *et al*. Disentangling the Effects of Water Stress on Carbon Acquisition, Vegetative Growth, and Fruit Quality of Peach Trees by Means of the QualiTree Model. *Front. Plant Sci*. **9** (2018). 10.3389/fpls.2018.00003 PMC5788000 29416545

[22] Ding N (2017). Effects of crop load on distribution and utilization of 13C and 15N and fruit quality for dwarf apple trees. Sci. Rep..

[23] Bussi C, Génard M (2014). Thinning and Pruning to Overcome Alternate Bearing in Peach Trees. Eur. J. Hortic. Sci..

[24] Bellingeri M, Quilot-Turion B, Oliveira Lino L, Bevacqua D (2018). The Crop Load Affects Brown Rot Progression in Fruit Orchards: High Fruit Densities Facilitate Fruit Exposure to Spores but Reduce the Infection Rate by Decreasing Fruit Growth and Cuticle Cracking. Front. Ecol. Evol..

[25] Bussi C, Plenet D, Merlin F, Guillermin A, Mercier V (2015). Limiting brown rot incidence in peach with tree training and pruning. Fruits.

[26] Gibert C (2009). Modelling the effect of cuticular crack surface area and inoculum density on the probability of nectarine fruit infection by Monilinia laxa. Plant Pathol..

[27] Bevacqua D, Quilot-Turion B, Bolzoni L (2018). A model for temporal dynamics of brown rot spreading in fruit orchards. Phytopathology.

[28] Bertin N, Génard M (2018). Tomato quality as influenced by preharvest factors. Sci. Hortic. (Amsterdam)..

[29] De Swaef T (2014). Model-assisted evaluation of crop load effects on stem diameter variations and fruit growth in peach. Trees - Struct. Funct..

[30] Lescourret F, Moitrier N, Valsesia P, Génard M (2011). QualiTree, a virtual fruit tree to study the management of fruit quality. I. Model development. Trees.

[31] Mirás-Avalos JM (2013). Assessment of the water stress effects on peach fruit quality and size using a fruit tree model, QualiTree. Agric. Water Manag..

[32] Gilligan C, van den Bosch F (2008). Epidemiological models for invasion and persistence of pathogens. Annu. Rev. Phytopathol..

[33] Gubbins S, Gilligan CA, Kleczkowski A (2000). Population dynamics of plant-parasite interactions: thresholds for invasion. Theor. Popul. Biol..

[34] Mailleret L, Grognard F (2009). Global stability and optimisation of a general impulsive biological control model. Math. Biosci..

[35] Cunniffe N, Gilligan C (2010). Invasion, persistence and control in epidemic models for plant pathogens: the effect of host demography. J. R. Soc. - Interface.

[36] Mercier V, Bussi C, Plenet D, Lescourret F (2008). Effects of limiting irrigation and of manual pruning on brown rot incidence in peach. Crop Prot..

[37] Mercier V, Bussi C, Lescourret F, Génard M (2009). Effects of different irrigation regimes applied during the final stage of rapid growth on an early maturing peach cultivar. Irrig. Sci..

[38] Bussi C, Parveaud CE, Mercier V, Lescourret F (2016). Effects of irrigation deprivation and ground cover (Trifolium repens) in the tree row on brown rot incidence in peach. Crop Prot..

[39] Brown PH, Weinbaum SA, Picchioni GA (1995). Alternate bearing influences annual nutrient consumption and the total nutrient content of mature pistachio trees. Trees Struct. Funct..

[40] Besset J, Génard M, Girard T, Serra V, Bussi C (2001). Effect of water stress applied during the final stage of rapid growth on peach trees (cv. Big-Top). Sci. Hortic. (Amsterdam)..

[41] Gibert C, Chadœuf J, Vercambre G, Génard M, Lescourret F (2007). Cuticular cracking on nectarine fruit surface: spatial distribution and development in relation to irrigation and thinning. J. Am. Soc. Hortic. Sci..

[42] Mirás-Avalos JM (2011). QualiTree, a virtual fruit tree to study the management of fruit quality. II. Parameterisation for peach, analysis of growth-related processes and agronomic scenarios. Trees.

[43] Emery KM, Michailides TJ, Scherm H (2000). Incidence of latent infection of immature peach fruit by Monilinia fructicola and relationship to brown rot in Georgia. Plant Dis..

[44] Alcobendas R, Mirás-Avalos J, Alarcón JJ, Nicolás E (2013). Effects of irrigation and fruit position on size, colour, firmness and sugar contents of fruits in a mid-late maturing peach cultivar. Sci. Hortic. (Amsterdam)..

[45] Mirás-Avalos J (2016). Using midday stem water potential for scheduling deficit irrigation in mid–late maturing peach trees under Mediterranean conditions. Irrig. Sci..

[46] Ould-Sidi MM, Lescourret F (2011). Model-based design of integrated production systems: a review. Agron. Sustain. Dev..

[47] Grechi I (2012). Designing integrated management scenarios using simulation-based and multi-objective optimization: Application to the peach tree–*Myzus persicae* aphid system. Ecol. Modell..

[48] Thomas ARC, Bond AJ, Hiscock KM (2013). A multi-criteria based review of models that predict environmental impacts of land use-change for perennial energy crops on water, carbon and nitrogen cycling. GCB Bioenergy.

[49] Caruso T, Guarino F, Bianco RL, Marra FP (2015). Yield and profitability of modified Spanish bush and Y-trellis training systems for peach. HortScience.

[50] Marini RP (2003). Peach Fruit Weight, Yield, and Crop Value Are Affected by Number of Fruiting Shoots per Tree. HortScience.

[51] Nelsen, R. *An Introduction to Copulas*. *Springer Publishing Company* (Springer New York, 2010).

[52] Box G, Wilson K (1951). Journal of the Royal Statistical Society. J. R. Stat. Soc. Ser. B.

