# Supplementary material for: Coupling epidemiological and tree growth models to control fungal diseases spread in fruit orchards
Source: Sci Rep. 2019 Jun 11;9:8519. doi: 10.1038/s41598-019-44898-6 (PMC6560096; doi:10.1038/s41598-019-44898-6)
Supplement: Supplementary file 1 — Results-Table [file 41598_2019_44898_MOESM1_ESM.pdf]

Authors: Bevacqua D.[1], Génard M.[1] , Lescourret F.[1] , Martinetti D.[2], Vercambre G.[1] , Valsesia P.[1] , Mirás-Avalos J. M. [1]

1: UR 1115, Plantes et Systèmes de Culture Horticoles, Institut National de la Recherche Agronomique, Avignon, France. 2: UR 546, Bistatistique et Processus Spatiaux, Institut National de la Recherche Agronomique, Avignon, France. Correspondence and requests for materials should be addressed to D.B. (email: daniele.bevacqua@inra.fr)

Title: Coupling epidemiological and tree growth models to control fungal diseases spread in fruit orchards.

Table 1: Management scenario (MS) characterized by crop load (CL (fruit tree^{-1})) and the value of stem water potential at midday, at the fruit growth period *j* (RDI_j). Estimated yield per plant in absence of disease (Y), and in presence of the disease, hence structured into susceptible (Y_S), exposed (Y_E) and infected fruit (Y_I), average fruit fresh mass (*m*) and sweetness (s) at harvest, monetary value per unit of mass yield (MV). See the text for details on the computation of presented values.

MS	CL	RDI_I	RDI_{II}	RDI_{III}	Y(kg)	Y_S(kg)	Y_E(kg)	Y_I(kg)	*m* (g)	s (%)	MV
1	53	-0.7	-0.7	-0.7	11.03	10.87	0.01	0.05	208	5.9	0.25
2	157	-0.7	-0.7	-0.7	30.91	18.46	2.95	6.26	197	5.7	0.22
3	471	-0.7	-0.7	-0.7	74.91	0.91	2.22	31.21	159	4.8	0.10
4	53	-0.7	-1.4	-0.7	10.82	10.73	0.01	0.03	204	5.9	0.25
5	157	-0.7	-1.4	-0.7	30.35	26.64	1.10	1.79	193	5.7	0.22
6	471	-0.7	-1.4	-0.7	73.08	3.71	10.77	38.83	155	4.8	0.10
7	53	-0.7	-2.2	-0.7	10.59	10.52	0.01	0.02	200	5.8	0.25
8	157	-0.7	-2.2	-0.7	29.68	27.28	0.73	1.13	190	5.7	0.21
9	471	-0.7	-2.2	-0.7	71.10	6.94	15.97	34.95	151	4.8	0.10
10	53	-0.7	-0.7	-1.4	6.93	6.85	0.01	0.02	131	9.0	0.72
11	157	-0.7	-0.7	-1.4	18.97	14.82	1.33	1.75	121	8.9	0.64
12	471	-0.7	-0.7	-1.4	42.48	2.01	5.58	17.67	90	8.3	0.30
13	53	-0.7	-0.7	-2.2	4.66	4.61	0.01	0.01	86	12.3	0.35
14	157	-0.7	-0.7	-2.2	12.91	10.41	0.88	0.92	82	12.5	0.29
15	471	-0.7	-0.7	-2.2	29.56	23.89	3.34	1.41	63	11.8	0.14
16	53	-0.7	-1.4	-1.4	6.81	6.77	0.00	0.01	128	9.0	0.71
17	157	-0.7	-1.4	-1.4	18.68	18.08	0.20	0.23	119	8.9	0.62
18	471	-0.7	-1.4	-1.4	41.54	35.17	3.90	1.91	88	8.4	0.29
19	53	-0.7	-1.4	-2.2	4.55	4.53	0.00	0.00	86	12.3	0.33
20	157	-0.7	-1.4	-2.2	12.64	12.54	0.03	0.03	81	12.5	0.28
21	471	-0.7	-1.4	-2.2	28.15	28.03	0.04	0.00	60	12.1	0.12
22	53	-0.7	-2.2	-1.4	6.63	6.59	0.00	0.01	125	9.0	0.68
23	157	-0.7	-2.2	-1.4	18.18	17.77	0.14	0.15	116	8.8	0.59
24	471	-0.7	-2.2	-1.4	40.27	36.49	2.34	1.09	86	8.4	0.26
25	53	-0.7	-2.2	-2.2	4.46	4.44	0.00	0.00	84	12.5	0.31
26	157	-0.7	-2.2	-2.2	12.25	12.17	0.02	0.02	78	12.5	0.25
27	471	-0.7	-2.2	-2.2	27.26	27.14	0.04	0.00	58	12.1	0.11
28	53	-1.4	-0.7	-0.7	10.97	10.84	0.01	0.04	207	5.9	0.25
29	157	-1.4	-0.7	-0.7	30.76	22.87	2.14	3.91	196	5.7	0.22
30	471	-1.4	-0.7	-0.7	74.42	1.37	3.89	35.44	158	4.8	0.10
31	53	-1.4	-1.4	-0.7	10.77	10.68	0.01	0.02	203	5.9	0.25
32	157	-1.4	-1.4	-0.7	30.24	26.96	0.98	1.58	193	5.7	0.22

33	471	-1.4	-1.4	-0.7	72.63	4.44	12.30	38.18	154	4.8	0.10
34	53	-1.4	-1.4	-1.4	6.76	6.73	0.00	0.01	128	9.0	0.70
35	157	-1.4	-1.4	-1.4	18.60	18.07	0.18	0.20	118	8.9	0.62
36	471	-1.4	-1.4	-1.4	41.25	35.04	3.81	1.86	88	8.4	0.28
37	53	-1.4	-1.4	-2.2	4.52	4.50	0.00	0.00	85	12.3	0.33
38	157	-1.4	-1.4	-2.2	12.58	12.49	0.03	0.02	80	12.5	0.27
39	471	-1.4	-1.4	-2.2	27.94	27.82	0.04	0.00	59	12.1	0.12
40	53	-1.4	-2.2	-0.7	10.52	10.45	0.01	0.02	199	5.8	0.24
41	157	-1.4	-2.2	-0.7	29.55	27.20	0.71	1.11	188	5.6	0.21
42	471	-1.4	-2.2	-0.7	70.80	7.07	16.10	34.60	150	4.8	0.10
43	53	-1.4	-2.2	-1.4	6.58	6.54	0.00	0.01	124	9.0	0.67
44	157	-1.4	-2.2	-1.4	18.07	17.68	0.13	0.15	115	8.8	0.58
45	471	-1.4	-2.2	-1.4	39.98	36.28	2.29	1.06	84	8.3	0.26
46	53	-1.4	-2.2	-2.2	4.45	4.44	0.00	0.00	84	12.6	0.31
47	157	-1.4	-2.2	-2.2	12.17	12.09	0.02	0.02	77	12.5	0.25
48	471	-1.4	-2.2	-2.2	27.04	26.92	0.04	0.00	57	12.1	0.11
49	53	-1.4	-0.7	-1.4	6.89	6.86	0.00	0.00	130	9.0	0.72
50	157	-1.4	-0.7	-1.4	18.90	16.99	0.66	0.79	120	8.9	0.63
51	471	-1.4	-0.7	-1.4	42.19	25.52	9.54	5.57	89	8.4	0.30
52	53	-1.4	-0.7	-2.2	4.63	4.61	0.00	0.00	87	12.3	0.35
53	157	-1.4	-0.7	-2.2	12.84	12.47	0.13	0.12	82	12.5	0.29
54	471	-1.4	-0.7	-2.2	28.81	28.68	0.04	0.00	61	12.1	0.13
